# Comparison of art preferences in patient rooms between chronic pain patients, palliative care patients and physicians

**DOI:** 10.1007/s10354-025-01086-8

**Published:** 2025-05-05

**Authors:** Alina Balandin, Martin Wallenfang, Kerstin Wagener, Martin Gleim, Dag Konetzka, Dieter Siebrecht, Anke Müffelmann, Susanne Kollmann, Regina Göckede, Christiane Zippel, Almut Linde, Annette Hüsch, Stine Louring Nielsen, Markus Steinfath, Axel Fudickar

**Affiliations:** 1 Department of Anaesthesiology and Intensive Care Medicine, Campus Kiel, University Hospital Schleswig-Holstein, Arnold-Heller-Str. 3/R, 24105 Kiel, Germany; 2 Friedrich-Ebert-Krankenhaus Neumünster GmbH, Neumünster, Germany; 3 Klinikum Leer, Leer, Germany; 4 Kunsthalle zu Kiel, Christian-Albrechts-University, Kiel, Germany; 5 Muthesius University of Fine Arts and Design, Kiel, Germany; 6 Aalborg University, Department of Architecture, Design and Media Technology, Copenhagen, Denmark

**Keywords:** Evidence-based art, Evidence-based design, Art, Hospital, Health Care Design

## Abstract

**Background:**

Art in patient rooms can have positive effects on wellbeing and clinical outcomes. Patients’ art preferences may differ from the preferences of medical providers. In this study, chronic pain and palliative care patients’ preferences regarding art in patient rooms were compared with physicians’ preferences.

**Methods:**

From a selection of abstract art photographs by artists of the Muthesius University of Fine Arts and Design, Kiel, and figurative paintings from the Kunsthalle zu Kiel, 79 physicians and 79 chronic pain patients were asked to choose a picture. The physicians were also asked which picture they would choose for their patients. Furthermore, 79 palliative care patients were investigated to compare their choices with those of pain patients and physicians.

**Results:**

Both patient groups preferred figurative art more often than did physicians for their patients. Among palliative care patients, 65% chose figurative art, while only 42% of physicians did (*p* < 0.0001). Similarly, 67% of chronic pain patients preferred figurative art, in contrast to 42% of physicians (*p* = 0.0002). The incidence of physicians’ art choices for figurative art in patient rooms and for themselves as patients differed significantly (42 vs. 58, *p* = 0.013). Views with natural elements were preferred by physicians for patients and themselves (49 vs. 30 and 44 vs. 35), by palliative care patients (41 vs. 38), and by chronic pain patients (54 vs. 25).

**Conclusion:**

Patients’ art preferences differ significantly from physicians’ art preferences.

**Supplementary Information:**

The online version of this article (10.1007/s10354-025-01086-8) contains supplementary material, which is available to authorized users.

## Background

Visual art seems to mitigate stress caused by hospitalization [1]. However, artwork is generally not chosen by patients but rather by clinical staff, whose decisions may differ from patients’ preferences [2, 3]. Evidence-based art (EBA) involves the investigation of art effects [4, 5] and can guide the use of art in hospitals [6], since art can improve wellbeing, symptoms, and signs [7]. In a recent review, we found that patients’ preferences for nature scenes can be explained by the human tendency to be attracted to convenient nature (biophilia) [7, 8]. However, social interaction and avoidance of mental deprivation are enhanced by various types of artwork; thus, we recommend a diverse mix of abstract and figurative art for public rooms [20]. Nevertheless, the level of evidence of the assessed studies was low, and EBA may therefore not be considered when artwork is chosen [8].

**Fig. 1:**
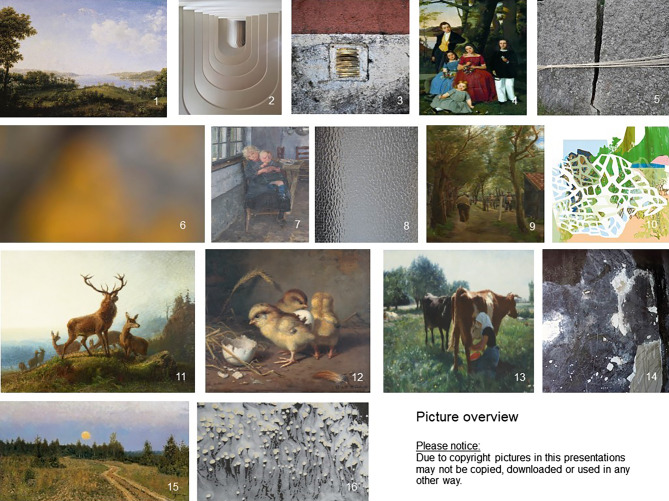
Artwork presentation: presentation of figurative and abstract artwork for interrogation of patients and physicians. Artists: 1. Johann Ludwig Hansen d. Ä., 2. Emilia Grone, 3. Helena Sachs, 4. Detlev Conrad Blunck, 5. Johannes Eggers, 6. Locu Ratolo, 7. Detlev Conrad Blunck, 8. Paula Oltmann, 9. Max Liebermann, 10. Meike Schlemmer, 11. Johann Christian Kröner, 12. Konrad Gustav Sues, 13. Hans Olde d.Ä. 14. Helena Sachs, 15. Isaak Lewitan, 16. Svenja Grossmann

The objective of this study was to compare art style preferences (Fig. 1) in patient rooms between chronic pain patients and physicians. Additionally, the art preferences of palliative care patients were exploratively compared with the art preferences of the first two groups. A preference for natural views was investigated exploratively in all groups.

## Methods

This prospective observational monocentric nonrandomized study was performed from April 2021 to November 2022 at a tertiary care center (German Clinical Trials Register, DRKS00019845, 03.01.2020; ethics committee approval on 03.12.2019; chair: Prof. H. M. Mehdorn, Christian-Albrechts-University Kiel, Germany; no. D563/19). All data were recorded anonymously. 79 adult chronic pain patients, 79 adult palliative care patients, and 79 physicians from the Department of Anesthesiology and Operative Intensive Care Medicine, University Hospital Schleswig-Holstein, Campus Kiel, were included. Patients without written informed consent, underage patients or patients unable to provide informed consent, and patients without communication ability were excluded.

After providing written informed consent, patients were asked in a verbal interview using a standardized questionnaire to choose one of 16 artworks that they would prefer for their patient room and to give reasons for their choice. Age, sex, and the highest educational grade were obtained. Physicians were anonymously asked in written form using a standardized questionnaire which of the same 16 artworks they would prefer for their patients’ rooms and which artwork they would prefer in their room if they were patients themselves, with the option to give reasons. Information about the study was presented to physicians during an educational meeting beforehand, and consent was given by anonymous participation.

The 16 artworks included eight figurative pictures from the 19th century provided by the Kunsthalle zu Kiel, Christian-Albrechts-Universität, Kiel, Germany, depicting landscapes, animals, and people and chosen according to favored motives in the literature [8] and according to motive preferences in our previous study on dream suggestions in anesthesia. This study was designed to investigate the effect of preoperative positive dream suggestion on the incidence and content of dreaming during general anesthesia. Patients selected their preferred dream content before the induction of general anesthesia and were verbally guided to relax and imagine these dreams. Commonly preferred dream contents were activities in nature, pets, and relatives or friends. Therefore, we assumed that patients would regard these motifs as appropriate subjects of figurative art in hospitals [9].

The eight figurative pictures were selected from 25 pictures preliminarily selected by R. Göckede, C. Zippel, and A. Hüsch. The abstract artworks were abstract photographs chosen from artworks created for hospitals by artists of the Muthesius University of Fine Arts and Design, Kiel, Germany, with written consent to use the works for the study and associated publications [10]. All artworks were presented on PowerPoint (Microsoft, Redmond, WA, USA) slides and on a wall poster.

### Statistics

On the basis of the data of a previous study [11], a group size of 79 participants per group was calculated to identify a difference of 0.2 in the incidence of a preference for figurative art with sufficient power (β = 0.8). Power analysis was performed via free statistical programs (HyLown Consulting LLC, Atlanta, USA, and Select Statistical Services, Exeter, UK [12, 13]). An α < 0.05 was regarded as statistically significant. Differences in the incidence of preferences for figurative art between palliative care patients and chronic pain patients and between palliative care patients and physicians were analyzed exploratively.

Incidences between groups were compared via Fisher’s exact test and the χ^2^ test. Demographic data were compared with the t‑test for normally distributed data and the Wilcoxon test for nonnormally distributed data. Data analysis was performed with Graph Pad Prism for Mac, version 5.03 (GraphPad Software, San Francisco, USA).

## Results

Between April 2019 and November 2022, 79 physicians, 79 adult chronic pain patients, and 79 adult palliative care patients from the Department of Anesthesiology and Operative Intensive Care Medicine, University Hospital Schleswig-Holstein, Campus Kiel, were interviewed for this prospective study.

### Patient characteristics

#### Demographics

The age of the chronic pain patients ranged from 22 to 87 years, and the mean age of these patients was 58.5 ± 16.4 years. The sex distribution was 48 women versus 28 men (3 patients provided no sex information in the questionnaire). The educational grades of chronic pain patients ranged from high school (1), university entrance qualification (2), middle school (3), and secondary school (4) to elementary school (5; median [range]: 3 [1–5]). The age of the palliative care patients ranged from 40 to 90 years, and the mean age of these patients was 68.0 ± 12.5 years. A total of 45 women and 34 men were included in the palliative care group. As with chronic pain patients, palliative care patients’ educational grades ranged from elementary school to a general qualification for university entrance, and the median educational grade was also secondary school leaving certificate (3 [1, 3], median [range]).

Age and sex distributions differed between palliative and chronic pain patients (68.0 years vs. 58.5 years, *p* = 0.0001, and 34 males/45 females vs. 28 males/48 females, *p* = 0.51, respectively). Educational grades were not different between these groups (*p* = 0.10), but the median educational grades of both groups differed from the physicians’ median grade (3 [1–5] vs. 1 [1–1], *p* < 0.0001). The demographic data are shown in Table 1.

**Table 1:** Demographic data

	Chronic pain patients	Palliative patients	*p-*value
Male/female^a^	28/48	34/45	0.51^h^
Age^b^	58.5 ± 16.4	68.0 ± 12.5	< 0.0001^i^
BMI^c^	28.3 ± 6.3	23.5 ± 4.7	< 0.0001^j^
ASA I/II/III/IV^d^	7/35/18/0	0/2/71/1	< 0.0001^k^
Education 1/2/3/4/5^ef^	6/7/25/17/2	19/4/22/12/18	0.10^l^
*Diagnosis*^*g*^			
Chronic back pain	16	–	–
Chronic pain, other	13	–	–
Fibromyalgia	10	–	–
Neuropathic pain	9	–	–
Polyneuropathy	7	–	–
Head and face pain	6	–	–
Other	18	–	–
GIT cancer	–	19	–
Lung cancer	–	12	–
Breast cancer	–	8	–
Urological cancer	–	8	–
Malignant melanoma	–	7	–
Other	–	20	–

Data are given as mean ± standard deviation or absolute numbers (percent)

ASA American Society of Anesthesiology physical status classification, *BMI *body mass index, *GIT* gastrointestinal tract

^a^*n* = 76/79

^b^*n* = 73/78

^c^*n* = 74/79

^d^*n* = 60/74

^e^*n* = 57/75 (number of chronic pain/palliative patients providing the information)

^f^Education: *1* high school diploma, *2* university entrance qualification, *3* middle school, *4* secondary school, *5* elementary school

^g^Most frequent diagnoses defined as diagnoses with a minimal incidence of 10% in at least one group

^h^Fisher’s exact test

^i^t‑test

^j^t‑test

^k^Fisher’s exact test (I, II, IV vs. III)

^l^Fisher’s exact test (1, 2, 4, 5 vs. 3)

### Preference for figurative or abstract art

**Fig. 2:**
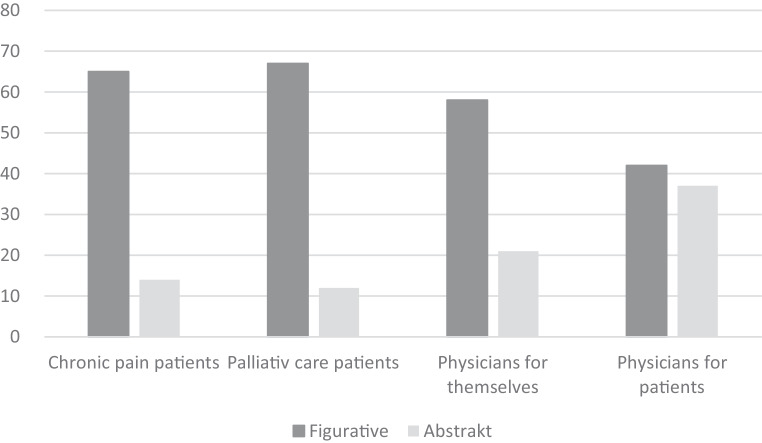
Picture choices of chronic pain patients, palliative care patients, physicians for themselves as patients, and physicians for their patients

Chronic pain patients preferred figurative art over abstract art significantly more often than did physicians (65 vs. 42, *p* < 0.0001). The same was shown significantly after post hoc Bonferroni correction for palliative care patients in comparison to physicians’ choices for their patients (67 vs. 42, *p* = 0.0002). Physicians’ art choices for figurative art in patient rooms and for themselves as patients also differed significantly after post hoc Bonferroni correction (42 vs. 58, *p* = 0.013); however, there was no difference between chronic pain and palliative care patients’ figurative art preferences (65 vs. 67, *p* = 1.0). There was also no significant difference between physicians’ figurative art choices for themselves as patients and palliative care patients’ and chronic pain patients’ figurative art choices (58 vs. 65, *p* = 0.25, and 58 vs. 67, *p* = 0.85, respectively; Fig. 2).

### Preference for natural views

Artworks were divided into artworks with predominantly natural views (1, 5, 10, 15) and others. All groups preferred pictures with elements of predominantly natural views of different extents (natural view vs. no natural view: palliative care patients 41 vs. 38; chronic pain patients 54 vs. 25; physicians for patients 49 vs. 30; and physicians for themselves 44 vs. 35).

### Different preferences as the most preferred figurative pictures

A difference between the art preferences of chronic pain patients, palliative care patients, and physicians regarding their most preferred figurative pictures was observed.

The two most favored motifs overall were number 15 and number 1, which are concordant with the biophilia hypothesis. The biophilia hypothesis, also known as the savannah hypothesis, suggests that humans have an innate affinity for landscapes featuring water, fertile vegetation, shade-providing trees, and open spaces.

**Fig. 3:**
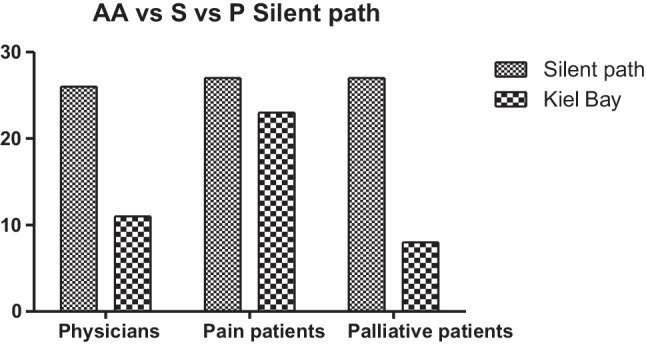
Picture choice for picture number 1 (Kiel Bay) and picture number 15 (Silent path) of chronic pain patients, palliative care patients, and physicians for their patients

There was a difference in the most preferred figurative pictures between the groups. Among palliative care patients, 27 participants preferred picture number 15, whereas only 8 chose picture number 1. However, 27 chronic pain patients selected picture number 15, while 23 chose picture number 1 (*p* = 0.008). A comparable distribution was observed among physicians selecting artwork for their patients, with 26 preferring picture number 15 and only 11 choosing picture number 1 (*p* = 0.0001; Fig. 3).

### Gender-specific differences

Male palliative care patients chose abstract art more often than female palliative care patients (abstract vs. figurative: male 14 vs. 29, female 0 vs. 35; *p* = 0.0001). There was also a difference between male and female pain patients that narrowly missed significance (abstract vs. figurative: male 6 vs. 20, female 3 vs. 47; *p* = 0.055).

### Patients’ and physicians’ comments on their art choice

The 125 comments were categorized into “calming effect” (*N* = 29), “pleasing image composition” (*N* = 18), “conveys security” (*N* = 1), “pleasing motive” (*N* = 25), “cognitive reflections on positive effects” (*N* = 12), “pleasing nature scene” (*N* = 24), and “general positive impression” (*N* = 1; Table 2; all comments provided as supplementary data).

**Table 2:** Categorization of the comments

	Chronic pain patients	Palliative patients	Physicians for their patients	Physicians for themselves as patients	Summarized number of comments
Calming effect	18	2	4	5	29
Pleasing image composition	0	0	12	6	18
Conveys security	0	1	0	0	1
Pleasing motive	8	5	6	6	25
Cognitive reflections on positive effects	4	3	1	4	12
Pleasing nature scene	17	0	4	3	24
General positive impression	7	0	4	5	16
Summarized number of comments	54	11	31	29	125

Patients’ comments on their art choice were categorized. The number of comments is given for each category in Table 2, together with the summarized number of comments for each category and each group.

## Discussion

Main results:

1. Chronic pain patients choose figurative artwork for their patient rooms significantly more often than physicians would have done for their patients.
2. There was no difference in the incidence of figurative art preferences between chronic pain patients and palliative care patients, nor between physicians choosing for themselves as patients. However, the art choices of physicians for patient rooms and for themselves as patients differed significantly.
3. Patients and physicians preferred artwork that included elements of natural views.
4. There was a difference between chronic pain patients’, palliative care patients’, and physicians’ art preferences regarding their most preferred figurative pictures.

### Preference for figurative art

The preference for figurative art confirms our recent review on EBA [8]. An explanation for this preference may be that abstract art may enhance negative emotions by associating abstract structures with negative content related to hospitalization [6, 14, 15]. Favorable figurative artwork may distract cognition from uncomfortable feelings [16]. However, the preference for figurative art may have been biased by the observation that preferences for figurative artworks coincide more often between different viewers than preferences for abstract artworks, as in our study [17].

### Preference for abstract art

Few patients but nearly half of the physicians chose abstract art for their rooms or their patients’ rooms, which corresponded to the variety of art preferences in general [18, 19]. In two studies, abstract art may have had a positive effect on patient wellbeing, similar to that of figurative art [20, 21]. This notion has been explained by the different possible interpretations of abstract paintings [11]. Abstract art might also promote positive coping with disease through mental openness [22] and might meet patients’ demands for stimulating sensual experiences related to their cultural background [22, 23]. Ambiguous structures in abstract art can encourage reassessment and the development of a new, positive mental framework [24].

Abstract art can also act by focusing on color effects, due to the great importance of picture colors for patient preferences [21]. According to this observation, some Danish hospital art projects, such as the design of a hospital in Herlev by Poul Gernes, emphasize the positive effects of color [25].

### Discordance between physicians’ choices for their patients and physicians’ choices for themselves

Physicians’ art choices for patient rooms and their choices for themselves as patients differed significantly. Physicians may have chosen abstract art for their patients because of the possibility of different interpretations, thus avoiding motifs that may have displeased their patients. Moreover, they may have preferred abstract art while working in patient rooms or may have regarded abstract art as a motivation for patients to reflect on their situation. Abstract art may encourage patients to think about both the artwork and their situation, serving as a potentially challenging yet effective means of psychosocial recovery. Although this reflection may initially cause discomfort and rejection compared to the comfort offered by pleasing figurative landscapes, it may serve as a long-term remedy by promoting coping strategies for the disease and its sequelae.

### Preference for natural motives

Most favored motives are concordant with biophilia, the theory of which claims that humans prefer safe and convenient areas for living in nature [7, 14]*. *Accordingly, window views on nature have been shown to positively influence recovery after surgery [26], and nature views can effectively distract from stressful events such as examinations or surgical procedures under local anesthesia [27].

### Comparison of art preferences between chronic pain and palliative care patients

Overall, there was no difference between the art preferences of chronic pain and palliative care patients. However, while palliative patients favored a melancholic view with dawn over a forest (15), most chronic pain patients preferred a serene summer view over a bay (1). Following the theory of paradoxical positive emotion, the esthetic experience reflecting their inner state may have positive effects on the wellbeing of palliative care patients by inducing positive emotions despite their incurable disease.

### Various art forms in public areas

Composite abstract and figurative art in social areas and public rooms is regarded as appropriate for meeting the needs of all patients, enhancing social interaction and preventing mental deprivation [8, 18, 19, 22]. The results of our study also suggest a relationship between art preference and educational level. Thus, a mixed approach in public areas may also meet the preferences of differently educated patients, visitors, and hospital staff.

### Limitations

Scientific methods have been regarded as inadequate for investigating art effects because of their complexity [4]*. *However, the quantifiable interrogatory psychometric investigations applied in our study can be applied to complex subjective phenomena and can be analyzed by statistical methods, as is known, e.g., from pain research. Hence, scientific investigation of art effects is not only possible but also mandatory if artwork is claimed to support wellbeing and healing [30].

In our study, the masterworks of an art museum were compared with contemporary artworks from artists at a university of fine art and design who have not yet been proven successful by more than one century of art critics. Thus, quality could have confounded the measured effect of figurative versus abstract art [31]. However, the masterworks were not very famous, and the unverified novelty of the abstract artworks in our study did not deter physicians from choosing modern works in nearly half of the cases.

The observed difference between male and female chronic pain patients is interesting. However, it cannot be conclusively assessed because it was not a target criterion of the study. A confirmatory study would be required to make definitive statements. However, these results align with public and media discussions about general gender preferences, where differences in art preferences also seem to exist, although these are rarely addressed in scientific research [32–34]. According to this notion, women tend to prefer figurative art, while men are more inclined toward abstract art. However, these preferences are likely shaped by a complex interplay of cultural, social, and individual factors, suggesting that gender-specific art preferences are not solely determined by biological sex but are instead influenced by various psychosocial confounding factors.

## Conclusion

Chronic pain and palliative care patients choose figurative artwork for their rooms significantly more often than physicians choose figurative artwork for their patients’ rooms.

There were no differences in art preferences between chronic pain patients, palliative care patients, and physicians for themselves as patients. Patients and physicians generally prefer artworks including elements and colors of natural views, thus rendering figurative art with natural views especially apt for patient and intervention rooms due to their high acceptance. The highly individual preference for some abstract artworks in our study and the theoretical considerations support the notion that abstract art is more apt for public areas and can enhance coping and patient interaction.

## Supplementary Information

Table 3: Physicians comments on their picture choice

## Data Availability

All data supporting the results can be obtained from the corresponding author upon request.

## References

[1] Harris PB, McBride G, Ross C, et al. A place to heal: environmental sources of satisfaction among hospital patients. J Appl Soc Psych. 2002;32:1276–99. 10.1111/j.1559-1816.2002.tb01436.x.

[2] Sigler M. Über Wandschmuck in Arztpraxen und ein tragisches Missverständnis. Tintenklex Interdiszip Z Nachwuchswiss. 2017;1:104–8.

[3] Danko D. Kunstsoziologie. Bielefeld: transcript; 2012.

[4] Fich LB, Hansen CØ, Nielsen SML, et al. Kunstens Potentiale i Sundhedsvæsnet; Hospitaler: Valg af kunstnerisk udsmykning; Proces og metode. Aalborg: Institut for Arkitektur; 2016.

[5] Hamilton DK. The four levels of evidence-based practice. Healthc Des. 2003;3:18–26.

[6] Ulrich R. Effects of viewing art on health outcomes. In: Frampton SB, Charmel PA, Planetree, editors. Putting patients first: best practices in patient-centered care. New York: Jossey-Bass; 2009. pp. 129–49.

[7] Lankston L, Cusack P, Fremantle C, et al. Visual art in hospitals: case studies and review of the evidence. J R Soc Med. 2010;103:490–9. 10.1258/jrsm.2010.100256. 21127332 10.1258/jrsm.2010.100256 PMC2996524

[8] Fudickar A, Konetzka D, Nielsen SL, Hawthorn K. Evidence based art in the hospital. Wien Med Wochenschr. 2022;172(9–10):234–41. 10.1007/s10354-021-00861-7. 34338902 10.1007/s10354-021-00861-7 PMC8326640

[9] Schäfer B, Klose P, Wiltfang J, Fudickar A. Incidence of dreaming and subjective quality of general anesthesia with and without standardized stress relaxation and dream suggestion by the anesthesiologist: a prospective Interventional randomized trial using an adaptive design. Dreaming. 2023;33(3):264–74. 10.1037/drm0000215.

[10] The vernissage of Muthesius Kunsthochschule Kiel https://muthesius-kunsthochschule.de/2019/04/18/vernissage-das-klinische-bild-kunst-befluegelt-genesung/. Accessed 19 Feb 2025.

[11] Nielsen SL, Fich LB, Roessler KK, et al. How do patients actually experience and use art in hospitals? The significance of interaction: a user-oriented experimental case study. Int J Qual Stud Health Wellbeing. 2017;12:1–11. 10.1080/17482631.2016.1267343. 10.1080/17482631.2016.1267343 PMC5328392 28452607

[12] HyLown Consulting LLC. http://powerandsamplesize.com/Calculators/Compare-2-Proportions/2-Sample-Equality.

[13] Select Statistical Services. Exeter EX1 3LH https://select-statistics.co.uk/calculators/sample-size-calculator-two-proportions/.

[14] Ulrich R, Gilpin L. Healing arts—nutrition for the soul. In: Frampton SB, Gilpin L, Charmel PA, editors. Putting patients first: designing and practicing patient-centered care. San Francisco: Jossey-Bass; 2003. pp. 117–46.

[15] Bower GH. Mood and memory. Am Psychol. 1981;36:129–48. 10.1037/0003-066X.36.2.129. 7224324 10.1037//0003-066x.36.2.129

[16] Fernandez E. A classification system of cognitive coping strategies for pain. Pain. 1986;26:141–51. 10.1016/0304-3959(86)90070-9. 3531980 10.1016/0304-3959(86)90070-9

[17] Vessel EA, Rubin N. Beauty and the beholder: highly individual taste for abstract, but not real-world images. J Vision. 2010;10:1811–4. 10.1167/10.2.18. 10.1167/10.2.18 PMC3662030 20462319

[18] Staricoff R, Lopper S. Integrating the arts into health care: can we affect clinical outcomes? In: Kirklin D, Richardson R, editors. The healing environment; without and within. London: Royal College of Physicians; 2003.

[19] Tovborg A, Ruge AM, Doran E, et al. Hvad gør kunst på hospitaler? In: Kunsten.nu. 2019. https://artmatter.dk/artguide/calendar/goer-kunst-paa-hospitaler. Accessed 19 Feb 2025.

[20] Nielsen SL, Mullins MF, Fich LB, et al. The significance of certain elements in art for patients’ experience and use. Vis Anthropol. 2017;30:310–27. 10.1080/08949468.2017.1333360.

[21] Moss H, O’Neill D. The aesthetic and cultural interests of patients attending an acute hospital—a phenomenological study. J Adv Nurs. 2013;70:121–9. 10.1111/jan.12175. 23731005 10.1111/jan.12175

[22] Serritzlew AD. Kunstens potentiale i sundhedsvæsnet – Hvordan kan kunstoplevelsen støtter patienter og pårørende i deres aktuelle udfordringer under ophold på Righospitalets patienthotel. Socialmedicinsk Tidskr. 2019;2:200–10.

[23] World Health Organization European health report. More than numbers—evidence for all. In: Data and Evidence WHO Regional Office for Europe; 2018.

[24] Rollins JA. Arousing curiosity: when hospital art transcends. Herd. 2011;4(3):72–94. 10.1177/193758671100400306. 21866505 10.1177/193758671100400306

[25] Gernes US, Hornung PM. Farvernes medicin – Poul Gernes og Amtssygehuset i Herlev. København: Borgen Forlag; 2005.

[26] Kaplan R, Kaplan S. The experience of nature: a psychological perspective. New York: Cambridge University Press; 1989.

[27] Reber R, Schwarz N, Winkelman P. Processing fluency and aesthetic pleasure: is beauty in the perceiver’s processing experience? Pers Soc Psychol Rev. 2004;8:364–82. 10.1207/s15327957pspr0804_3. 15582859 10.1207/s15327957pspr0804_3

[28] Hanson H, Schroeter K, Hanson A, et al. Preferences for photographic art among hospitalized patients with cancer. Onc Nurs Forum. 2013;40:E337–E45. 10.1188/13.ONF.E337-E345. 10.1188/13.ONF.E337-E345 23803278

[29] Han K. Responses to six major terrestrial biomes in terms of scenic beauty, preference, and restorativeness. Environ Behav. 2007;39(4):529–56. 10.1177/0013916506292016.

[30] Baum M. Book of the month: the healing environment: without and within. J Royal Soc Med. 2004;97:145–6. 10.1258/jrsm.97.3.145.

[31] Nanda U, Eisen SL, Baladandayuthapani V. Undertaking an art survey to compare patient versus student art preferences. Environ Behav. 2008;40(2):269–230. 10.1177/0013916507311552.

[32] Salkind L, Salkind NJ. Gender and age differences in preference for works of art. Stud Art Educ. 1997;38(4):246–56. 10.2307/1320524.

[33] Mueller S. Gender and art appreciation: sex makes a difference. Medical economics 2015 february 5 https://www.medicaleconomics.com/view/gender-and-art-appreciationsex-makes-a-difference. Accessed 19 Feb 2025.

[34] Tröndle M, Kirchberg V, Tschacher W. Subtle differences: men and women and their art reception. J Aesthetic Educ. 2014;48(4):65–93. 10.5406/jaesteduc.48.4.0065.

