# Supplementary material for: Comparison of art preferences in patient rooms between chronic pain patients, palliative care patients and physicians
Source: Wien Med Wochenschr. 2025 May 5;175(9-10):237–44. doi: 10.1007/s10354-025-01086-8 (PMC12089170; doi:10.1007/s10354-025-01086-8)
Supplement: Supplementary file 1 — Table 3: Physicians comments on their picture choice [file 10354_2025_1086_MOESM1_ESM.docx]

Supplement

Table 3: Physicians comments on their picture choice

| Picture number | Physicians for their patients | Physicians for themselves as patients |
| --- | --- | --- |
| 1 | - Pictures with nature/landscape have attested a calming effect - Quietness, nature, water - Reassuring - At least bright, friendly, regional motive - View can wander in the far, one stands safe on the viewers position, the water is in the far, the colors let the situation seem warm, friendly. - Neutral, appropriate for different age groups, relation to Kiel | - Pictures with nature/landscape have attested a calming effect - Quietness, nature, water - View in the far (hopeful) - Elicits childhood memories - Quite bright and friendly. While viewing it, one can find distraction in the thought, what can be seen there today - The viewer stands raised, can look at everything in quietness. I connect positive memories with water (river, sea), e.g. swimming, sailing, summer, easiness; where water is a river, North-/Baltic Sea is always something in motion, there is no stagnation |
| 2 | - Abstract, modern, clear form, bright - Calm, graphic pattern, no obtruding allegory |  |
| 3 | - Clear forms in proportioning/composition, hence sufficient structure and variety to render prolonged viewing exciting | - Clear forms in proportioning/composition, hence sufficient structure and variety to render prolonged viewing exciting - neutral, no relation to sickness/death to recognize |
| 4 |  |  |
| 5 |  |  |
| 6 | - Unexcited, modern, good contrast |  |
| 7 |  |  |
| 8 |  |  |
| 9 |  |  |
| 10 | - Friendly, colourful, vivid - Bright and friendly | - Friendly, colourful, vivid - Friendly, calming, detailed, distracting, stimulating fantasy |
| 11 |  |  |
| 12 |  |  |
| 13 |  |  |
| 14 |  |  |
| 15 |  | - Exudes calmness |
| 16 |  |  |

Table 4: Patients comments on their picture choice

| Picture number | Chronic Pain Patients | Palliative Care Patients |
| --- | --- | --- |
| 1 | - Beautiful calm nature to relax - Wideness, sky, calming - Nice warm sun, strand, light-flooded forest - Nature - Because it is spacious, beautiful weather, nice landscape - Blue sky, friendly, brightening - The view, I like the nature and water, calming - Calming - Nature is something beautiful calming, picture colours could be brighter - The picture exudes calmness, wideness and serenity - Calming effect - I like forest and water very much - I can imagine that I sojourn there and enjoy nature. The view provides relaxation and seduces to dream - Open wide positive - Exudes quietness, sun/daylight rises mood, realistic, you can imagine yourself very well in this place - Nice view, good weather, Nature`s quietness, proximity to water - Picture from romantic, exudes much calmness - Nature - Light and friendly, not so "unhappy", mediation of positive mindset - Nice summer day in nature |  |
| 2 | - The picture appears calming |  |
| 3 |  | - Appeals patient, modernity - Something to think about |
| 4 | - Colourful design, vitality, life, shows people, care - Family | - Family picture |
| 5 |  |  |
| 6 | - Soothing warmness - Light at the end of the tunnel | - Wide, agreeable colors, remembrance to beautiful experiences |
| 7 | - Realistic, scene, you can picture yourself in - I know this picture, bought postcard with this motive in Denmark and enjoyed to send them | - Concealment, protection |
| 8 |  |  |
| 9 | - Feeling of concealment, the alley and the depicted century make you calmer and more relax - Beautiful pied colors, invites to interpretation - Colorful, art |  |
| 10 |  |  |
| 11 | - Natural and animals - Nature and vivid - Bright sky, strong deer, has a calming effect on me - Joy of life - The picture is vivid | - Harmony, quietness |
| 12 | - I like it, good for my soul, calming - Because it looks sweet - Fresh life, like animals, mostly the chicken | - Animal love |
| 13 | - Relaxation | - Patients comes from rural area, brings back good memories |
| 14 |  |  |
| 15 | - Exudes calmness - One doesn`t know, where the path goes, is there a house? Animals? - On the one hand calming effect, on the other hand opportunity to discover something new - Allegorical rising sun, new beginning - Gives quietness and energy, other pictures are depressive - Nature, relaxing - Relaxing - Harmonic, calming, full of mood, space for interpretation - Calming me - Has a relaxing effect on me - Calming me - Nature |  |
| 16 |  | - One sees every time something else |
